# Parasiticidal Properties of Nanoemulsion-Based Plant Essential Oil Formulations for Controlling Poultry Ectoparasites in Farm Conditions

**DOI:** 10.3390/insects15110829

**Published:** 2024-10-23

**Authors:** Jarongsak Pumnuan, Anuwat Lakyat, Ampon Klompanya, Duangkamol Taemchuay, Amorn Assavawongsanon, Thanaporn Doungnapa, Somsak Kramchote

**Affiliations:** 1 School of Agricultural Technology, King Mongkut’s Institute of Technology Ladkrabang, Bangkok 10520, Thailand; anuwatraky@gmail.com (A.L.); ampon.kl@kmitl.ac.th (A.K.); duangkamol.ta@kmitl.ac.th (D.T.); somsak.kr@kmitl.ac.th (S.K.); 2 Faculty of Agricultural Technology, Rajamangala University of Technology Thanyaburi, Thanyaburi, Pathum Thani 12130, Thailand; amorn_a@rmutt.ac.th; 3 Thailand Institute of Scientific and Technological Research (TISTR), Khlong Luang, Pathum Thani 12120, Thailand; k.thanapornmilk@gmail.com

**Keywords:** clove, cinnamon, battery cage system, floor pen system, ornamental chicken

## Abstract

Nanoemulsion-based plant essential oil formulations (NEOFs) have shown remarkable parasiticidal properties in laboratory settings, suggesting the potential for practical farm applications. This study investigates the efficacy of NEOFs—comprising clove and cinnamon essential oils (EOs)—in controlling poultry ectoparasites under real farm conditions. We evaluated the impact of NEOFs on ectoparasite populations, egg-laying performance, egg quality, leukocyte profiles in chicken blood, and potential insecticide residues in eggs. Conducted across various poultry farms, the results revealed that NEOFs achieved an over 95% ectoparasite eradication after two treatments, compared to significantly higher ectoparasite populations in untreated control groups. Egg production was notably higher in NEOF-treated flocks, and the feed conversion ratio was improved. No significant differences in leukocyte profiles were observed between the treated and control groups. In contrast, cypermethrin residues were detectable in eggs for more than eight weeks post-treatment. NEOFs achieved an over 97% ectoparasite extermination within seven days post-treatment, with farmer satisfaction averaging 4.83 out of 5.00. These findings position NEOFs as a highly effective, environmentally friendly alternative to chemical pesticides for managing poultry ectoparasites, offering a viable solution for sustainable poultry farming.

## Simple Summary

This study evaluates nanoemulsion-based plant essential oil formulations (NEOFs) containing clove and cinnamon oils for controlling poultry ectoparasites in farm conditions. The results showed that NEOFs achieved an over 95% ectoparasite eradication after two treatments, compared to significantly higher levels in untreated controls. NEOF-treated flocks had increased egg production and improved feed conversion ratios, with no significant changes in leukocyte profiles. Unlike cypermethrin, NEOFs left no detectable residues. These findings highlight NEOFs as a highly effective and eco-friendly alternative to chemical pesticides, promoting sustainable poultry farming practices.

## 1. Introduction

Ectoparasites, including insects, ticks, and mites, are significant pests in poultry farming, attacking poultry by sucking blood or feeding on the skin, feathers, or scales. These infestations lead to several adverse effects, such as retarded growth, reduced liveliness, discomfort, allergies, and dermatitis. Ectoparasites directly impact the quality and quantity of poultry meat and eggs, and indirectly act as vectors for dozens of pathogens [1,2]. For instance, Erdem et al. [3] reported that poultry red mite infestations are significant stress factors, with severe infestations leading to considerable mortality rates. These data confirm that mite infestations can affect egg production, egg quality, and feed conversion in poultry [4,5]. Some lice cause anemia by feeding on blood, resulting in skin inflammation and eventually feather loss [6]. Chickens are infected with anemia due to these blood-sucking parasites. Al-Saffar and Al-Mawla [7] reported an increase in total white blood cells, especially heterophils and eosinophils, in chickens infected with ectoparasites compared to a non-infected control group. Ticks and mites are responsible for these hemorrhagic changes, whereas flies and most lice do not cause anemia in birds. The study by Wamboi et al. [8] showed that poultry parasites are prevalent and contribute to altered haemato-biochemical parameters, such as a decrease in total erythrocyte count and an increase in total leukocyte count.

The current problems present a major impediment in poultry farms and the poultry industry worldwide [9,10]. Previously, reports have indicated that mite infestations have caused significant economic losses in Europe, affecting millions of hens and leading to severe economic losses in the egg production industry [11,12]. In Thailand, Lakyat et al. [13] reported that mites and lice are the most destructive external parasites in poultry farms, particularly in egg-laying and ornamental chicken farms in the eastern area of Bangkok. Ectoparasites are more prevalent in extensive farming systems than in intensive farming systems and more common in exotic chickens than in local breeds [9]. Currently, the main treatments available against poultry ectoparasites in farm conditions are synthetic pesticides (insecticides and acaricides) such as organophosphates, pyrethroids, carbamates [14], and isoxazolines [15]. However, the application of synthetic pesticides in poultry farms negatively impacts consumers of poultry products. Pesticides can accumulate in the fatty tissue of animals [16], and have been detected in various animal products, including meat and eggs [17]. Marangi et al. [14] reported pesticide residues in laying hens from poultry farms in Southern Italy, showing the presence of carbaryl and permethrin in chicken organs, tissues, and eggs.

Similarly, Túri et al. [18] found propetamphos and permethrin residues in meat samples from chickens in Hungary. Abdelfatah and Abu-Zeid [19] reported dimethoate pesticide residues in eggs samples from the El-Sharkia Province, Egypt, exceeding the maximum residue limits (MRLs). Hamid et al. [20] noted bifenthrin insecticide residues in egg samples from poultry farms, also exceeding the MRLs, posing potential risks to humans.

Resistance to pesticides in ectoparasite infections is continually increasing, leading to the use of more toxic chemicals [21]. Resistance has been reported in ectoparasites such as *Sarcoptes scabiei*, *Rhipicephalus microplus*, and *Varroa destructor* [22]. Knolhoff and Onstad [23] reported that pyrethroids, organophosphate, and carbamate insecticides, commonly used for controlling veterinary pests, act as synaptic nerve poisons. Pyrethroid resistance can occur through target site mutations in sodium channels, leading to knock-down resistance. Organophosphate and carbamate resistance usually occurs through metabolic detoxification or the presence of insensitive acetylcholinesterase.

In addition to the continuous problems from chemical pesticide applications, environmental concerns are raised regarding the use of pesticides on livestock [23,24]. Many farms avoid pesticide use to adhere to organic farming standards or to meet consumer demands for chemical-free poultry products [25]. Therefore, exploring environmentally friendly alternatives to replace chemical insecticides in poultry farm conditions is urgently necessary. Bioactive compounds from medicinal plants have been used as parasiticides due to their relatively low impact on animal and human health and the environment. Essential oils (EOs) from various plants have shown a high efficacy in controlling poultry ectoparasites [26,27,28]. EOs from ginger (*Zingiber officinale*) and citronella (*Cymbopogon nardus*) have demonstrated a high effectiveness against lice (*Menopon gallinae*) and mites (*Ornithonyssus bursa*) [29]. EOs from pennyroyal (*Mentha pulegium*) have shown toxicity against the poultry red mite (*Dermanyssus gallinae*) [30], 2009). Tea tree oil (*Melaleuca alternifolia*) has been evaluated against chewing lice (*Bovicolao cellatus*), showing high parasiticidal activity and causing the complete mortality of sheep lice ([31,32]. EOs from clove buds (*Syzygium aromaticum*), cinnamon leaves (*Cinnamomum zeylanicum*), and turmeric rhizomes (*Curcuma longa*) have effectively killed lice (*Lipeurus caponis*) [33]. Lakyat et al. [34] demonstrated the parasiticidal properties of clove, cinnamon, and turmeric EOs in the form of NEOFs for controlling poultry ectoparasites. NEOFs of clove and cinnamon EOs have exhibited excellent parasiticidal activities, making them suitable for farm applications. However, most medicinal plants’ effectiveness as parasitical products has been confirmed in laboratory conditions but needs further validation in the field.

The objective of this research was to evaluate the parasiticidal properties of NEOFs for controlling poultry ectoparasites under farm conditions. Additionally, to assess the egg-laying performance and egg quality of chickens, the type and quantity of leukocytes in chicken blood, and insecticide residues in eggs after using the NEOFs to control poultry ectoparasites in farm conditions. The effectiveness of NEOFs in ornamental chicken farms was also included in this study.

## 2. Materials and Methods

### 2.1. Preparation of Nanoemulsions-Based Plant Essential Oil Formulations (NEOFs)

Essential oils (EOs) were obtained from dried clove buds (*Syzygium aromaticum*) and dried cinnamon leaves (*Cinnamomum zeylanicum*). The selection of these was based on previous laboratory research by Pumnuan et al. [33]. The EOs were obtained from the Thai-China Flavors and Fragrances Industry Co. Ltd. (Bangkok, Thailand) and prepared according to principles of hazard analysis and critical control point (HACCP). From the major compounds analysis of both EOs by gas chromatography mass spectrometer (GC-MS) (Agilent Technologies Inc., CA, USA), it was found that eugenol was found to be the predominant compound in clove and cinnamon EOs, constituting 85 and 75% of the oils, respectively. Nanoemulsions of clove and cinnamon EOs (NEO-CL and NEO-CN, respectively) or NEOs were prepared following the methodology described by Doungnapa et al. [35]. Each NEO was prepared with EO/Tween 60/PEG 400 ratios of 2:9:2. The NEOFs were mixtures of various NEOs in different ratio, NEOF-1 (NEO-CL:NEO-CN = 1:0) and NEOF-2 (NEO-CL:NEO-CN = 1:1), and the working solution of the NEOFs were prepared at 0.25% of EOs in water. The control was a mixture of surfactants, Tween60/PEG400 = 9:2 at 1.625% of surfactants in water, and a positive control was prepared from cypermethrin insecticide 35% EC at the recommended dose (0.1% in water). The particle size and polydispersity index (PDI) of the NEOF-1 and NEOF-2 formulations were measured using a Nano plus Zeta/Nano Particle Analyzer (Micromeritics Instrument Corporation; Osaka, Japan) with the manufacturer’s software; the formulations exhibited particle sizes of 20 to 21 nm and PDIs of 0.30–0.31.

### 2.2. Effectiveness of NEOFs for Controlling Poultry Ectoparasites in Egg-Laying Chicken Farm

As previously presented by Lakyat et al., [34] reported the parasiticidal properties of clove and cinnamon EOs in the form of NEOFs for controlling poultry ectoparasites in the laboratory. These formulas demonstrated exceptional potency in exterminating ectoparasites, with an LC_50_ and LC_90_ at <0.160 and <0.250%, respectively, 6 h after treatments. Therefore, their effectiveness as parasitical NEOF products of clove and cinnamon EOs made them suitable for farm applications.

**Figure 1:**
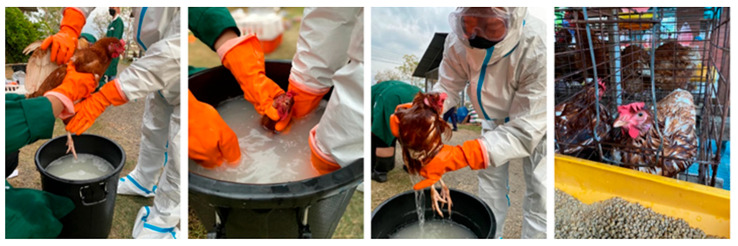
The dipping method: hens were dipped in the solution, covering the entire body for 1 min, then picked up and placed in a cage.

The efficacy of NEOFs against ectoparasites was assessed in farm conditions at the Smart Chicken Farm, School of Agricultural Technology, King Mongkut’s Institute of Technology Ladkrabang (KMITL), Thailand. The systems of the chicken farm, including battery cages and floor pen systems, were evaluated with 35-week-old laying hens. Both systems were maintained in environmental control houses with 5 h of ventilation fan operation and a 16 h lighting program per day. All treatments provided *ad libitum* food and water, with a food mix formula suitable for laying hens during the egg-laying stage. In the battery cage system, hens were raised in single cages, while in the floor pen system, hens were raised free on the floor (cage size 3 m^2^, 10 hens per cage). The experiment followed a randomized complete block design with five randomized replicates, each comprising 4 hens for the battery cages system (a total of 20 hens per treatment). For the floor pen system, two randomized replicates were used, each with 10 hens (total 20 hens per tretment). Five treatments were tested over 12 weeks: NEOF-1 (0.25% of EO in water), NEOF-2 (0.25% of EO in water), cypermethrin insecticide (0.1% in water), control (1.625% of surfactants in water), and blank (water). These treatments were prepared in 30 L of water, and placed in a 32 L plastic bucket (diameter 28 cm, height 52 cm). Each laying hen was dipped in the solution for 1 min, then placed in cages according to the experimental plan (Figure 1). This test was carried out twice: at 35 and 41 weeks old, respectively. Mites and lice are major ectoparasites on the chickens and were randomly counted before treatment (day 0), and on days 1, 3, 5, 7, and 14 after treatments (1st and 2nd times). Ectoparasites were collected from 5 random points of each chicken body (neck, wing, breast, back, and buttocks) following methods adapted from Al-Saffar and Al-Mawla [7] and Lakyat et al. [13]. In total 20 feathers were randomly collected from around the neck (4 feathers), on the wings (4 feathers), in the front of the breast (4 feathers), the back (between the cape) (4 feathers), and around the anus (4 feathers). The type and quantity of all ectoparasites found in each experiment were classified in the laboratory.

### 2.3. Egg-Laying Performance and Quality of Chicken Eggs After Using the NEOFs to Control Poultry Ectoparasites in an Egg-Laying Chicken Farm

The egg-laying performance and quality of chicken eggs were evaluated alongside the study of the effectiveness of NEOFs against ectoparasites under farm conditions, as mentioned earlier. These evaluations followed protocols described by Dao et al. [36]. The percentages of hen-day egg production (%Hen-day), egg weight, and egg mass were recorded daily. The %Hen-day was calculated by multiplying the total number of eggs laid by 100 and dividing that by the product of the number of days and the number of hens alive on each of those days. Feed consumption was recorded weekly, and the feed conversion ratio (FCR) was calculated by dividing feed intake by egg mass.

### 2.4. Type and Quantity of Leukocytes in Chicken Blood After Using NEOFs to Control Poultry Ectoparasites in an Egg-Laying Chicken Farm

Hematology tests on chicken blood were conducted in this research to assess the impact of NEOFs on poultry ectoparasites under farm conditions. The type and quantity of leukocytes in chicken blood cells were evaluated before treatment and 7 days after the second treatment. Leukocytes in the blood were categorized into two groups: granulocyte (heterophil, eosinophil, and basophil), and agranulocyte (lymphocyte and monocyte). Blood smears were fixed in methanol and stained with Wright-Giemsa stain for a differential leukocyte count determination, according to the methodology described by Jain et al. [37].

### 2.5. Insecticide Residue in Eggs After Using NEOFs to Control Poultry Ectoparasites in an Egg-Laying Chicken Farm Compared with Insecticide Application

The assessment took place of insecticide residue in egg samples after using NEOFs to control poultry ectoparasites under farm conditions, compared with traditional insecticide applications. In this study, three eggs of each replication were randomly sampled before treatment (day 0), and 1, 3, 5, 7, and 14 days after the first and second treatments. The extraction and quantitative analysis of cypermethrin insecticide in the egg samples were performed using modified QuEChERS methods [38,39]. Cypermethrin insecticide residue (CIR) was analyzed using gas chromatography (GC) (Agilent Technologies Inc., CA, USA). The GC apparatus was equipped with a DB-5MS capillary column (30 m length × I.D. 0.32 mm × 0.25 µm film thickness). The analysis parameters included the direct injection of 1 µL in splitless mode, with an injection temperature of 250 °C. Helium was used as a carrier gas at a flow rate of 2 mL/min. The oven temperature started at 125 °C, held for 1 min, then increased by 25 °C/min until reaching 250 °C, after which it was raised by 2 °C/min until it reached 280 °C. The detector temperature was maintained at 300 °C.

### 2.6. Effectiveness of NEOFs to Control Poultry Ectoparasites in Ornamental Chicken Farm

The effectiveness of NEOFs for controlling poultry ectoparasites was assessed in seven ornamental chicken farms located in the Pathum Thani province and Bangkok, Thailand. The ornamentals raised in these farms included Silkie chickens and Thai bantam chickens. Two methods were used in these experiments: the dipping method and the direct spray method. The dipping method used NEOFs at the same concentration and process as the test in the egg-laying chicken farm mentioned previously. For the direct spray method, a 0.25% concentration of NEOFs was used, with 15 mL sprayed all over each chicken. Ectoparasites on the chickens were randomly counted before treatment (0 days) and at 3 and 7 days after treatment. The classification of ectoparasites for each experiment followed the same methods as those used in the egg-laying chicken farm tests adapted from Al-Saffar and Al-Mawla [7] and Lakyat et al. [13].

The satisfaction level with the NEOF products was evaluated through a survey that assessed the product characteristics and usage characteristics and the effectiveness of NEOFs in controlling poultry ectoparasites in ornamental chickens. A 5-point Likert scale was adopted for data collection [40], measuring satisfaction levels with NEOF product qualities as follows: 1 = Highly unsatisfied, 2 = Unsatisfied, 3 = Neutral, 4 = Satisfied, and 5 = Highly satisfied. Additional suggestions were also collected to serve as a guideline for developing future formulations.

### 2.7. Data Analysis

The experiment assessing the effectiveness of NEOFs for controlling poultry ectoparasites in egg-laying chicken farms was carried out using a randomized complete block design (RCBD) with five randomized replicates in the battery cage system. The floor pen system used two randomized replicates. In the ornamental chicken farms, a completely randomized design was used with three randomized replicates. The data on egg-laying performance, chicken eggs quality, and the quantity of leukocytes in chicken blood after using NEOFs were statistically analyzed using an analysis of variance (ANOVA). Differences between the treatments were tested using Duncan’s multiple range test.

## 3. Results

### 3.1. Effectiveness of NEOFs for Controlling Poultry Ectoparasites in Egg-Laying Chicken Farm

The NEOFs were proven to effectively control poultry ectoparasites (Table 1). These formulations controlled 73.5–89.4% of ectoparasites in egg-laying chicken farms after the first treatment (leaving 10.6–26.5%) and demonstrated exceptional potency in exterminating more than 95% of ectoparasites after the second treatment. In the blank groups (water), the number of ectoparasites continuously increased, showing 2–5 times more ectoparasites than before treatment. In the positive control group (cypermethrin insecticide treatment), a significant reduction in ectoparasites was observed by day 3 after the first treatment. Interestingly, the control group (surfactant mixed with co-surfactant) controlled parasites to a certain extent, achieving more than 85% control in the battery cage system, although ectoparasites increased by 1.5 times after the second treatment compared to before the treatment. The prevalence of ectoparasites varied between farming systems, with the battery cage system showing a higher prevalence of ectoparasites than the floor pen system.

The prevalence of ectoparasites in egg-laying chickens before and after treatment with NEOFs is shown in Table 2. The results indicated that 11 species of ectoparasites were found attacking chickens before treatment. The mites *Megninia ginglymura* and *Pterolichus obtusus* were highly prevalent, with counts of 326.7 and 113.5 per random set, respectively. The lice *Lipeurus caponis* and *Menopon gallinae* also showed a high prevalence, with counts of 1418.3 and 323.4 per random set, respectively. The NEOFs (NEOF-1 and NEOF-2) showed a high performance against poultry ectoparasites in egg-laying chicken farms. These NEOFs achieved complete killing against *M. ginglymura*, *Megninia ortari*, *Ormithonyssus bursa*, *Ornithonyssus sylviarum*, *Cuclotogaster heterogoraphus*, *Goniocotes gallinae*, *Menacanthus stramineus* and *Menacanthus pallidulus*. They also showed more than 98% efficacy against *P. obtusus*, *L. caponis,* and *M. gallinae* 14 days after the second treatment.

### 3.2. Egg-Laying Performance of Chickens and Quality of Chicken Eggs After Using the NEOFs to Control Poultry Ectoparasites in Egg-Laying Chicken Farm

The egg production of laying hens from the blank group (water) showed a lower performance compared to the treatment groups. In contrast, the feed conversion (FCR) of the treatment groups was higher compared to the blank group (Table 3). The egg-laying performance and egg quality after using the NEOFs to control poultry ectoparasites showed no significant difference compared to the other treatments after the first application. After the second treatment, the performance of the NEOF groups did not significantly differ from the cypermethrin insecticide and control groups, except the blank group. The %Hen-day value in the treatments group was higher than the blank group, while the FCR value was lower. In the battery cage system, Hen-day production for all treatment groups ranged from 56.00% to 59.52%, which was significantly higher than that of the control group (47.41%) (*F* = 3.12, *df* = 4, *p* = 0.038). Similarly, in the floor pen system, Hen-day production for all treatment groups ranged from 47.13% to 49.57%, while the control group had a significantly lower production of 36.67% (*F* = 8.12, *df* = 4, *p* = 0.021). The FCR values in all treatment groups in the battery cage system and floor pen system (2.67–3.16 and 3.48–3.86, respectively) were significantly lower than those of the blank group (3.60 and 4.66, respectively) (battery cages system: *F* = 6.12, *df* = 4, *p* = 0.002; floor pen system: *F* = 15.48, *df* = 4, *p* = 0.005). Interestingly, the average egg weight from all treatments, including the blank group, showed no significant difference, with weights ranging from 45.17 to 49.91 g.

### 3.3. Type and Quantity of Leukocytes in Chicken Blood After Using NEOFs to Control Poultry Ectoparasites in Egg-Laying Chicken Farms

**Figure 2:**
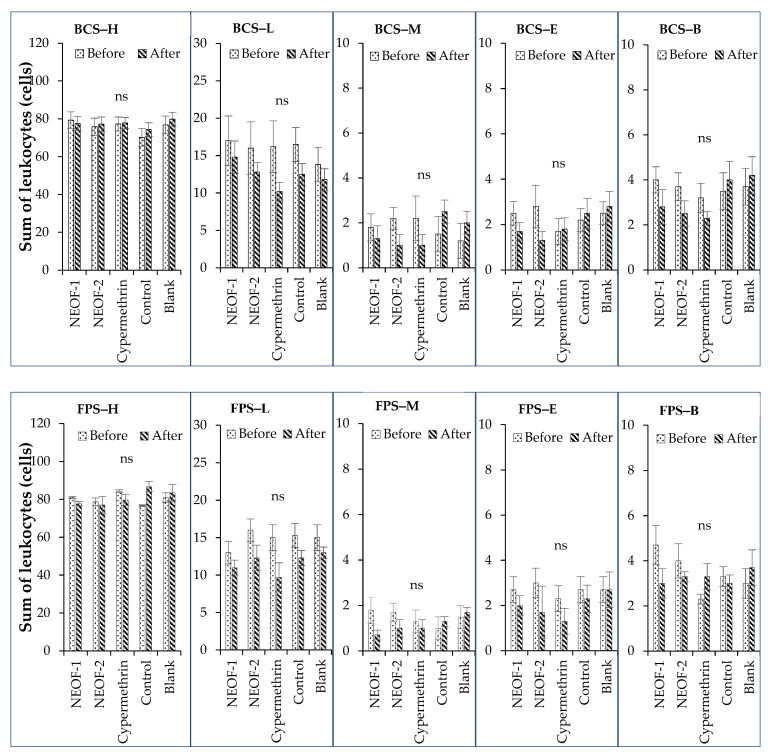
Differential leukocyte counts (means ± SE) in chicken blood after using nanoemulsion-based plant essential oil formulations (NEOFs) to control poultry ectoparasites in egg-laying chicken farm conditions (battery cases and floor pen systems) compared with cypermethrin insecticide application. Control (Tween60/PEG400 = 9:2, 1.625%), cypermethrin insecticide 35%EC (recommended dose; 0.1%), NEOF-1 (NEO-CL:NEO-CN = 1:0), NEOF-2 (NEO-CL:NEO-CN = 1:1), NEO-CL (clove/Tween60/PEG400 = 2:9:2), NEO-CN (cinnamon/Tween60/PEG400 = 2:9:2), NEOF-1 and NEOF-2 at 0.25% of EOs in water, BCS = battery cages system, FPS = floor pen system, H = heterophil, L = lymphocyte, M = monocyte, E = eosinophil, B = basophil, ns indicates non-significant difference at *p* < 0.05.

The type and quantity of leukocytes in chicken blood after using NEOFs to control poultry ectoparasites under farm conditions are shown in Figure 2. The results indicate that there were no significant differences in the qualities of each leukocyte type before and after treatment across all treatments. This lack of significant difference was observed in both the battery and floor pen systems. In all treatments, the highest average quantity of leukocytes was heterophil, ranging from 70 to 80 cells. This was followed by lymphocytes (10–17 cells), basophils (2–5 cells), eosinophils (1–4 cells), and monocytes (1–3 cells). These findings suggest that the use of NEOFs did not significantly alter the leukocyte profile in the chickens.

### 3.4. Insecticide Residue in Eggs After Using NEOFs to Control Poultry Ectoparasites in Egg-Laying Chicken Farms Compared with Insecticide Application

The cypermethrin insecticide residue (CIR) in egg samples after using NEOFs to control poultry ectoparasites in egg-laying chicken farms, compared with cypermethrin insecticide application, is shown in Table 4. In this study, CIR in the egg samples after using NEOFs was non-detectable (<0.002 ppm). In contrast, the egg samples after using cypermethrin insecticide showed no detectable residue on day 1 after treatment, but CIR was detected starting from day 3, with the highest quantities found on day 7. The residue levels then showed a decreasing trend but remained detectable for more than 8 weeks after treatment. Interestingly, CIR in egg samples from the battery cage system was higher (0.123 and 0.191 ppm) than in the floor pen system (0.106 and 0.117 ppm) on day 7 after the first and second treatments, respectively.

### 3.5. Effectiveness of NEOFs to Control Poultry Ectoparasites in Ornamental Chicken Farms

NEOFs, which were effective in controlling poultry ectoparasites in egg-laying chicken farms, also demonstrated a high efficacy in ornamental chicken farms (Table 5). The quantity of poultry ectoparasites was higher in Thai bantam chickens than in Silkie chicken across all ornamental chicken farms. The NEOFs showed a high control of ectoparasites in ornamental chickens, exterminating more than 83 and 97% of ectoparasites 3 and 7 days after treatment, respectively, for both direct spray and dipping methods. Both NEOF-1 and NEOF-2 were effective in controlling poultry ectoparasites.

The mean satisfaction scores ranged from 4.43 to 5.00, with an overall mean average of 4.83, indicating a very high value of satisfaction. The satisfaction ratings for product characteristics, usage characteristics, and the effectiveness of NEOFs in controlling poultry ectoparasites in ornamental chickens were 4.96, 4.81, and 4.71, respectively. Additionally, farmers suggested improvements for product development: (1) the direct spray method had limitations in effectively covering the whole body, (2) the dipping method may affect chicken feather color, and (3) the sticky nature of NEOFs could cause feathers to cling.

## 4. Discussion

The presence of ectoparasites in egg-laying chicken farms significantly impacts egg production. Our study demonstrated that a 3–5-fold increase in ectoparasite numbers correlated to lower %Hen-day values and a higher FCR, significantly different from the treated groups. Heavy ectoparasite loads can lead to health issues in hens, such as decreased egg production, increased mortality, and possibly anemia, affecting the productivity of the egg industry [4,41]. High ectoparasite loads induce stress, causing pain, skin irritation, aggressive feather-pecking, and cannibalistic behavior, leading to increased feed and water intake, decreased egg production, reduced egg quality, and poor overall animal health [4,42,43]. Ectoparasite infestations can result in a 20–30% drop in productivity [43,44,45].

The use of medicinal plants for controlling insects and mites has shown promising potential, with clove and cinnamon EOs being particularly effective. Previous studies have confirmed their efficacy against a wide range of pests, including those in crop plants [46], stored products [47], public health [48,49], pets [50], and livestock [33,51,52]. In addition, these EOs have demonstrated parasiticidal properties against poultry ectoparasites in the form of NEOFs in laboratory conditions [34]. This study extends those findings by confirming their efficacy under farm conditions.

Our results show that NEOFs effectively control poultry ectoparasites in egg-laying chicken farms, though their efficacy is somewhat lower compared to chemical pesticides. The first treatment with NEOFs controlled more than 75% of ectoparasites by day 3, whereas cypermethrin achieved over 95% control. The need for repeated applications of NEOFs to achieve similar levels of control suggests that the volatility of EOs, which leads to relatively short-lived parasiticidal activity, might be a limitation [50]. Incorporating surfactants can mitigate this issue by reducing the evaporation rate of EOs and enhancing their bioavailability and diffusion due to the wetting ability of surfactants [53]. This study showed that surfactants moderately enhanced the parasiticidal effectiveness, aligning with previous findings that they also exhibit insecticidal properties [54].

Contrary to some reports indicating an increase in total white blood cells in chickens infected with ectoparasites [7,8], our study found no significant difference in leukocyte levels across treatments. This may be due to the relatively low prevalence of ectoparasites in our study, which was insufficient to cause hematopoietic changes. Al-Saffar and Al-Mawla [7] noted that leukocytosis, heterophilia, and eosinophilia increased significantly in heavily infested birds. The disparity in ectoparasite types, with mites causing more significant changes than lice [7], could explain why our study, which found higher lice infestations, did not show significant hematologic changes. Consistently, heterophils were the most abundant leukocytes, while other hematologic cells were less prevalent, aligning with Wamboi et al. [8].

Consumer safety and product quality are paramount in agricultural practices. Despite the promotion of organic farming, chemical pesticide residues in agricultural products remain a concern [14,16,17,18,19,20]. Our study demonstrated that chemical pesticides used in egg production could result in residues persisting for more than 8 weeks post-treatment. Although the residue levels were low, they could accumulate in human tissues, posing risks such as cytotoxic disorders, immunotoxicity, hormonal changes, or carcinogenesis [55]. Furthermore, the dangers of pesticide exposure extend to farm workers involved in their application [55].

Nanoemulsion technology involves isotropic dispersions of two non-miscible liquids, oil and water, with particle sizes ranging from 100 to 400 nm. The selection of emulsifier type and formula ratios was carefully considered [48], and the polydispersity index (PDI) values of the nanoemulsions were maintained below 0.3, indicating a high stability [56]. In this study, NEOFs were optimized to the nano scale, with particle sizes of 20–21 nm and PDI values of 0.30–0.31. The acaricidal properties of essential oil (EO) nanoemulsions were found to be more effective against African red mites than EO emulsions [35]. Numerous studies have shown that combining essential oils results in a higher insecticidal efficacy than using pure EOs alone [57,58]. Thus, EO nanoemulsions in the form of NEOFs, composed of clove and cinnamon essential oils, are effective for controlling poultry ectoparasites in farm conditions.

NEOFs adapted for controlling ectoparasites in ornamental chickens showed high parasiticidal properties. Future modifications should ensure a comprehensive distribution throughout the animal to maximize exposure to active ingredients. NEOFs act as contact poisons, and their efficacy depends on their ability to bind to the lipid layers of insects and mites [59]. Surfactants in NEOFs reduce EO evaporation rates and enhance the induction efficiency of active ingredients into ectoparasites. Additionally, EOs exhibit insecticidal properties through fumigant action [60]. Ornamental chicken farmers showed a high satisfaction with NEOFs, although concerns about feather color and stickiness should be addressed in future formulations. These improvements are particularly important for ornamental chickens, where feather appearance significantly impacts their value.

Lice and mite ectoparasites remain on poultry throughout their lifespan, necessitating treatment by either spraying or dipping methods [61]. The direct spray method often proves less effective because the pressure does not ensure complete coverage or deep penetration to the skin where mites reside [62], and it also requires specialized equipment, increasing costs. Additionally, applying sprays to dry feathers may result in runoff, reducing the retention of the parasiticide. In contrast, the dipping method ensures wet feathers retain more of the active ingredient [61]. However, dipping can be labor-intensive and may induce stress in poultry. To mitigate stress, Campbell [61] suggests conducting treatments at night.

## 5. Conclusions

This study concludes that nanoemulsion-based plant essential oil formulations (NEOFs) containing clove and cinnamon essential oils are an effective and safer alternative to chemical insecticides for controlling poultry ectoparasites in egg-laying farms. NEOFs achieved an over 95% ectoparasite eradication after two treatments, comparable to cypermethrin, and showed no adverse effects on egg production, egg quality, or leukocyte profiles. Most importantly, NEOFs left no residues in eggs, highlighting their safety for human consumption and their potential as a sustainable solution for managing ectoparasites in poultry farming.

## Figures and Tables

**Table 1:** Percentages of all poultry ectoparasites per random set before and after treatment with nanoemulsion-based plant essential oil formulations (NEOFs) in egg-laying chicken farm conditions compared with cypermethrin insecticide application.

Treatments ^3^	Percentages of All Ectoparasites of Chicken per Random Set ^1^ Before and After Treatments											
	Before ^2^ Treated 1st	Weeks After First Treatment										
		-	-	-	1	2	6	-	-	-	7	8
		Days After Treated 1st					Before Treated 2nd	Days After Treated 2nd				
		1	3	5	7	14		1	3	5	7	14
Battery cages system												
NEOF-1	100.0 ^2^	59.7	22.8	19.7	25.2	17.6	52.7	13.0	<5	<5	<5	<5
NEOF-2	100.0	32.3	22.8	29.0	19.5	26.5	47.0	27.8	<5	<5	<5	<5
Cypermethrin	100.0	19.9	<5	<5	<5	<5	<5	<5	<5	<5	<5	<5
Control	100.0	57.3	35.5	46.1	32.0	41.1	88.5	59.2	37.7	16.3	16.8	14.4
Blank	100.0	101.3	121.5	95.7	121.7	137.6	<500	<500	<500	<500	<500	<500
Floor pen system												
NEOF-1	100.0	49.2	15.1	9.5	9.3	10.6	44.0	20.3	<5	<5	<5	<5
NEOF-2	100.0	16.4	11.8	13.7	8.9	11.2	30.0	14.7	<5	<5	<5	<5
Cypermethrin	100.0	15.1	<5	<5	<5	<5	<5	<5	<5	<5	<5	<5
Control	100.0	45.2	35.0	30.8	25.2	35.8	97.8	98.1	53.7	145.7	185.9	142.3
Blank	100.0	91.3	87.8	97.5	116.7	110.0	<500	<400	<200	<200	<300	<300

^1^ The random set is ectoparasites were taken randomly from 5 points of each chicken body such as the neck, wing, breast, back, and buttocks according to the methodology described by Lakyat et al. [13]. ^2^ All ectoparasites of chickens found before treated of 2197.5 ± 380.4 parasites per random set. ^3^ Control (Tween60/PEG400 = 9:2, 1.625%), cypermethrin insecticide 35%EC (recommended dose; 0.1%), NEOF-1 (NEO-CL:NEO-CN = 1:0), NEOF-2 (NEO-CL:NEO-CN = 1:1), NEO-CL (clove/Tween60/PEG400 = 2:9:2), NEO-CN (cinnamon/Tween60/PEG400 = 2:9:2), NEOF-1 and NEOF-2 at 0.25% of EOs in water.

**Table 2:** The prevalence of poultry ectoparasites (mites and lice) per random set on chicken body before treatment and 14 days after the second treatment with nanoemulsion-based plant essential oil formulations (NEOFs) in egg-laying chicken farm conditions.

Ectoparasites	Average of Ectoparasites on Chicken per Random Set ^1^		Percentages of Ectoparasite Mortality
	Before Treatment	14 Days After Second Treatment	
Mites			
*Megninia ginglymura*	326.7	0.0	100.0
*Megninia ortari*	2.4	0.0	100.0
*Ormithonyssus bursa*	0.3	0.0	100.0
*Ornithonyssus sylviarum*	1.2	0.0	100.0
*Pterolichus obtusus*	113.5	0.6	99.5
Total of mites	444.1	0.6	
Lice			
*Cuclotogaster heterogoraphus*	2.1	0.0	100.0
*Menopon gallinae*	323.4	2.4	99.3
*Lipeurus caponis*	1418.3	18.0	98.7
*Goniocotes gallinae*	7.8	0.0	100.0
*Menacanthus stramineus*	0.3	0.0	100.0
*Menacanthus pallidulus*	1.5	0.0	100.0
Total of lice	1753.4	20.4	
Total	2197.5	21.0	99.0

^1^ The random set is ectoparasites were taken randomly from 5 points of each chicken body such as the neck, wing, breast, back, and buttocks according to the methodology described by Lakyat et al. [13].

**Table 3:** Egg-laying performance of chickens and quality of chicken eggs after using nanoemulsion-based plant essential oil formulations (NEOFs) to control poultry ectoparasites in egg-laying chicken farm conditions (battery cases and floor pen systems) compared with cypermethrin insecticide application.

Treatments ^1^	After First Treatment				After Second Treatment			
	%Hen Day	Egg Weight (g)	Egg Mass (g)	FCR	%Hen Day	Egg Weight (g)	Egg Mass (g)	FCR
Battery cages system								
NEOF-1	61.95 ^a^	50.68 ^a^	31.12 ^a^	3.23 ^a^	56.00 ^a^	47.88 ^a^	26.81 ^ab^	2.67 ^b^
NEOF-2	63.71 ^a^	49.13 ^a^	31.28 ^a^	3.12 ^a^	57.74 ^a^	49.67 ^a^	28.67 ^a^	2.69 ^b^
Cypermethrin	61.71 ^a^	48.06 ^a^	29.70 ^a^	3.31 ^a^	59.52 ^a^	49.68 ^a^	29.48 ^a^	2.63 ^b^
Control	64.81 ^a^	48.17 ^a^	31.21 ^a^	3.28 ^a^	56.41 ^a^	49.91 ^a^	28.17 ^a^	3.16 ^ab^
Blank	60.00 ^a^	50.20 ^a^	30.09 ^a^	3.44 ^a^	47.41 ^b^	48.44 ^a^	22.96 ^b^	3.60 ^a^
Floor pen system								
NEOF-1	53.57 ^a^	51.15 ^a^	27.41 ^a^	5.86 ^a^	49.57 ^a^	48.50 ^a^	24.06 ^a^	3.48 ^b^
NEOF-2	57.14 ^a^	50.49 ^a^	28.94 ^a^	5.85 ^a^	49.93 ^a^	47.83 ^a^	23.89 ^a^	3.60 ^b^
Cypermethrin	52.86 ^a^	47.22 ^a^	24.95 ^a^	6.00 ^a^	47.95 ^a^	49.23 ^a^	23.61 ^a^	3.65 ^b^
Control	55.00 ^a^	49.90 ^a^	27.33 ^a^	6.96 ^a^	47.13 ^a^	48.65 ^a^	22.93 ^a^	3.86 ^b^
Blank	51.07 ^a^	46.23 ^a^	23.74 ^a^	7.40 ^a^	36.67 ^b^	45.17 ^a^	16.60 ^b^	4.66 ^a^

^1^ Control (Tween60/PEG400 = 9:2, 1.625%), cypermethrin insecticide 35%EC (recommended dose; 0.1%), NEOF-1 (NEO-CL:NEO-CN = 1:0), NEOF-2 (NEO-CL:NEO-CN = 1:1), NEO-CL (clove/Tween60/PEG400 = 2:9:2), NEO-CN (cinnamon/Tween60/PEG400 = 2:9:2), NEOF-1 and NEOF-2 at 0.25% of EOs in water. ^a,b^ Significantly different at *p* < 0.05.

**Table 4:** Cypermethrin insecticide residue in eggs after using nanoemulsion-based plant essential oil formulations (NEOFs) to control poultry ectoparasites in egg-laying chicken farm conditions (battery cases and floor pen systems) compared with cypermethrin insecticide application.

Treatments ^1^	Quantity of Cypermethrin Residues in Eggs (ppm)											
	Before Treated 1st	Weeks After 1st Treatment										
		-	-	-	1	2	6	-	-	-	7	8
		Days After 1st Treatment					Before Treated 2nd	Days After 2nd Treatment				
		1	3	5	7	14		1	3	5	7	14
Battery cages system												
Cypermethrin	Nd	Nd	0.048	0.101	0.123	0.104	0.029	0.030	0.102	0.173	0.191	0.153
Other treatments	Nd	Nd	Nd	Nd	Nd	Nd	Nd	Nd	Nd	Nd	Nd	Nd
Floor pen system												
Cypermethrin	Nd	Nd	0.060	0.085	0.106	0.061	0.026	0.022	0.062	0.101	0.117	0.073
Other treatments	Nd	Nd	Nd	Nd	Nd	Nd	Nd	Nd	Nd	Nd	Nd	Nd

^1^ Control (Tween60/PEG400 = 9:2, 1.625%), cypermethrin insecticide 35%EC (recommended dose; 0.1%), NEOF-1 (NEO-CL:NEO-CN = 1:0), NEOF-2 (NEO-CL:NEO-CN = 1:1), NEO-CL (clove/Tween 60/PEG 400 = 2:9:2), NEO-CN (cinnamon/Tween 60/PEG 400 = 2:9:2), NEOF-1 and NEOF-2 at 0.25% of EOs in water. Nd = not detected (<0.002 ppm), Other treatments include NEOF-1, NEOF-2, control, and blank groups.

**Table 5:** Average number of poultry ectoparasites on chickens per random set after using nanoemulsion-based plant essential oil formulations (NEOFs) to control poultry ectoparasites in ornamental chicken farms by direct spray and dipping methods.

Ornamental Chicken Breeds	Farms	Treatment ^2^	Average of Ectoparasites on Chicken per Random set ^1^		
			Before Treatment	Days After Treatment (%Decrease)	
				3	7
Direct spray method					
Thai bantam chicken	A	NEOF-1	51	0 (100.0)	1 (98.0)
		NEOF-2	32	21 (34.4)	0 (100.0)
	B	NEOF-1	95	9 (90.5)	0 (100.0)
		NEOF-2	119	14 (88.2)	5 (95.8)
	C	NEOF-1	46	11 (76.1)	0 (100.0)
		NEOF-2	36	6 (83.3)	0 (100.0)
	D	NEOF-1	137	26 (81.0)	1 (99.3)
		NEOF-2	88	11 (87.5)	1 (98.9)
Silkie chicken	A	NEOF-1	3	0 (100.0)	0 (100.0)
		NEOF-2	9	0 (100.0)	0 (100.0)
	E	NEOF-1	4	1 (75.0)	0 (100.0)
		NEOF-2	2	2 (0.0)	0 (100.0)
	F	NEOF-1	4	1 (75.0)	0 (100.0)
		NEOF-2	4	1 (75.0)	0 (100.0)
		Total	630	103 (83.7)	8 (98.7)
Dipping method					
Thai bantam chicken	A	NEOF-1	26	0 (100.0)	0 (100.0)
		NEOF-2	43	2 (95.3)	0 (100.0)
	E	NEOF-1	52	0 (100.0)	1 (98.2)
		NEOF-2	78	12 (84.6)	3 (96.2)
Silkie chicken	A	NEOF-1	20	0 (100.0)	0 (100.0)
		NEOF-2	7	2 (71.4)	0 (100.0)
	G	NEOF-1	5	2 (60.0)	0 (100.0)
		NEOF-2	9	2 (77.8)	3 (66.7)
		Total	240	20 (91.7)	7 (97.1)

^1^ The random set is ectoparasites were taken randomly from 5 points of each chicken body such as the neck, wing, breast, back, and buttocks according to the methodology described by Lakyat et al. [13]. ^2^ Control (Tween60/PEG400 = 9:2, 1.625%), cypermethrin insecticide 35%EC (recommended dose; 0.1%), NEOF-1 (NEO-CL:NEO-CN = 1:0), NEOF-2 (NEO-CL:NEO-CN = 1:1), NEO-CL (clove/Tween60/PEG400 = 2:9:2), NEO-CN (cinnamon/Tween60/PEG400 = 2:9:2), NEOF-1 and NEOF-2 at 0.25% of EOs in water.

## Data Availability

The data that support the findings of this study are available from the authors upon reasonable request.
